# Alternative mRNA Splicing in Three Venom Families Underlying a Possible Production of Divergent Venom Proteins of the Habu Snake, *Protobothrops flavoviridis*

**DOI:** 10.3390/toxins11100581

**Published:** 2019-10-09

**Authors:** Tomohisa Ogawa, Naoko Oda-Ueda, Kanako Hisata, Hitomi Nakamura, Takahito Chijiwa, Shousaku Hattori, Akiko Isomoto, Haruki Yugeta, Shinichi Yamasaki, Yasuyuki Fukumaki, Motonori Ohno, Noriyuki Satoh, Hiroki Shibata

**Affiliations:** 1 Department of Molecular and Chemical Life Science, Graduate School of Life Sciences, Tohoku University, Sendai, Miyagi 980-8577, Japan; ygthrsc05@gmail.com; 2 Department of Pharmaceutical Sciences, Faculty of Pharmaceutical Sciences, Sojo University, Kumamoto 860-0082, Japan; naoko@ph.sojo-u.ac.jp (N.O.-U.); nakamura@ph.sojo-u.ac.jp (H.N.); 3 Marine Genomics Unit, Okinawa Institute of Science and Technology Graduate University, Onna, Okinawa 904-0495, Japan; kanako@oist.jp (K.H.); norisky@oist.jp (N.S.); 4 Department of Applied Life Science, Faculty of Bioscience and Biotechnology, Sojo University, Kumamoto 860-0082, Japan; chijiwa@life.sojo-u.ac.jp (T.C.); mohno218@gmail.com (M.O.); 5 Institute of Medical Science, University of Tokyo, Oshima-gun, Kagoshima 894-1531, Japan; shattori@ims.u-tokyo.ac.jp; 6 Division of Genomics, Medical Institute of Bioregulation, Kyushu University, Fukuoka 812-8582, Japan; isomoto@gen.kyushu-u.ac.jp (A.I.); yfukumak@me.com (Y.F.); 7 DNA Sequencing Section, Okinawa Institute of Science and Technology Graduate University, Onna, Okinawa 904-0495, Japan.; shinichi.yamasaki@oist.jp

**Keywords:** venom genes and proteins, metalloproteinase, serine protease, vascular endothelial growth factor, transcriptome variants

## Abstract

Snake venoms are complex mixtures of toxic proteins encoded by various gene families that function synergistically to incapacitate prey. A huge repertoire of snake venom genes and proteins have been reported, and alternative splicing is suggested to be involved in the production of divergent gene transcripts. However, a genome-wide survey of the transcript repertoire and the extent of alternative splicing still remains to be determined. In this study, the comprehensive analysis of transcriptomes in the venom gland was achieved by using PacBio sequencing. Extensive alternative splicing was observed in three venom protein gene families, metalloproteinase (MP), serine protease (SP), and vascular endothelial growth factors (VEGF). Eleven MP and SP genes and a VEGF gene are expressed as a total of 81, 61, and 8 transcript variants, respectively. In the MP gene family, individual genes are transcribed into different classes of MPs by alternative splicing. We also observed trans-splicing among the clustered SP genes. No other venom genes as well as non-venom counterpart genes exhibited alternative splicing. Our results thus indicate a potential contribution of mRNA alternative and trans-splicing in the production of highly variable transcripts of venom genes in the habu snake.

## 1. Introduction

Many snakes have evolved the ability to produce venom, which is a complex mixture of toxic proteins. The toxic proteins contained in the venom are encoded by genes classified into more than ten gene families [1,2,3]. Protein components of venom are highly variable from species to species. Even within a species, these may vary from population to population and from individual to individual under different physiological and/or environmental conditions. This extensive diversity makes a full characterization of their repertoire difficult. However, recent venomics studies, including high-throughput transcriptomics (RNA-seq), have demonstrated a highly divergent profile of mRNAs encoded by venom genes, and the involvement of alternative splicing is suggested to contribute to transcriptome variety [4,5,6]. In the era of genome science, the extent of alternative splicing can be examined on a genome-wide scale.

In a previous study, we constructed a draft genome sequence of the habu snake, *Protobothrops flavoviridis* [7]. An approximately 1.4 Gbp genome assembly, HabAm1, was estimated to contain 25,134 protein-coding genes. A comprehensive annotation of habu genes with the aid of a large transcriptome dataset from 18 different organs has identified a total of 60 venom genes, which are categorized into 18 families [7]. These are metalloproteinase (MP), serine protease (SP), C-type lectin-like proteins (CTLP), phospholipase A_2_ (PLA2), three-finger toxins (3FTX), aminopeptidases (APaseN), cysteine-rich secretory proteins (CRISP), vespryns/SPla and ryanodine receptor domain proteins (Vespryn), 5′-nucleotidases (5Nase), dipeptidyl peptidases (DDPase), hyaluronidases (Hyal), nerve growth factors or neurotrophins (NGF), vascular endothelial growth factors (VEGF), L-amino acid oxidases (LAAO), phosphodiesterases (PDE), phospholipases B (PLB), bradykinin-potentiating peptides and C-type natriuretic peptides (BNP), and glutaminyl peptide cyclotransferases (GPCase). The first four constitute major families of habu venom proteins [3], including 11 gene copies of *svMP* (sv, snake venom) and *svSP*, 10 of *svCTLP,* and 9 of *svPLA2*. Multiple copies of these gene families were likely formed by multiple rounds of duplications of each of the original venom gene copies [7]. In addition to the accelerated evolution, alternative splicing has been reported as a diversifying mechanism in snake venom proteins such as acetylcholinesterases [8] and svSPs [9,10]. However, alternative splicing in venom protein genes has not been genome widely tested. In the present study, we have collected a large quantity of RNA-seq data obtained on a PacBio RSII platform, as well as by Illumina next-generation sequencing (NGS), for the annotation of HabAm1 genes. We describe details of transcripts of venom genes, carefully annotated by using Iso-Seq (isoform sequencing) long reads that span entire transcript isoforms, from the 5′ end to the 3′ polyA-tail. We also addressed the following questions: (1) How many venom genes exhibit alternative mRNA splicing? (2) how many splice variants are produced? and (3) by what mechanisms?

## 2. Results

### 2.1. Alternative Splicing Occurs in Three of the 18 Families of Venom Genes

**Figure 1:**
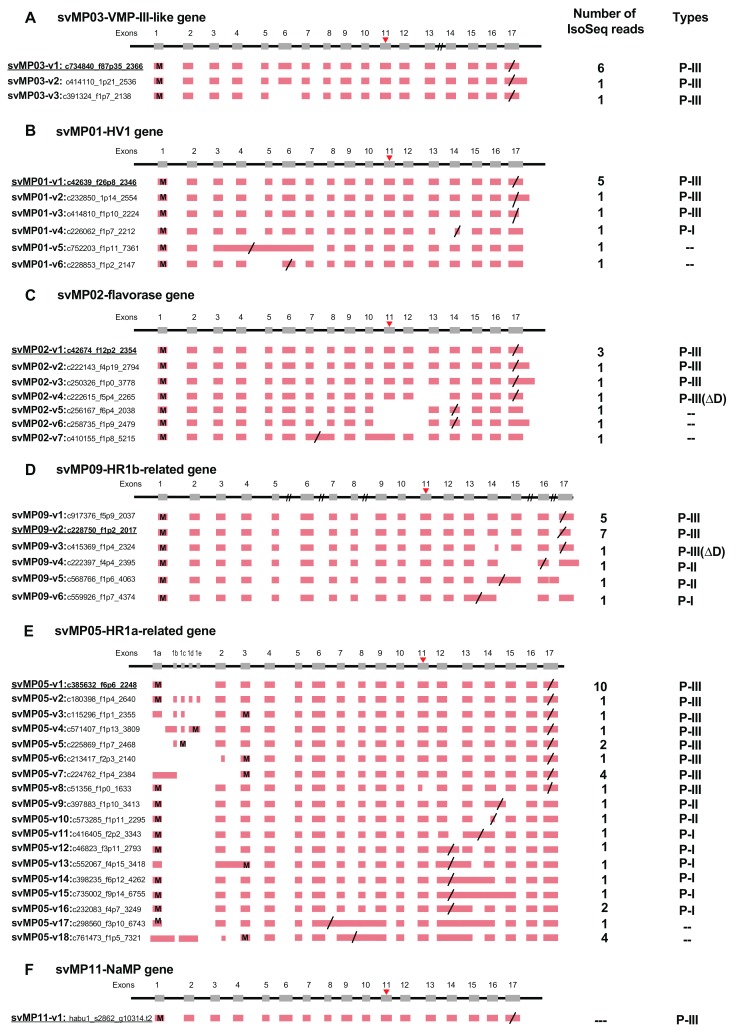

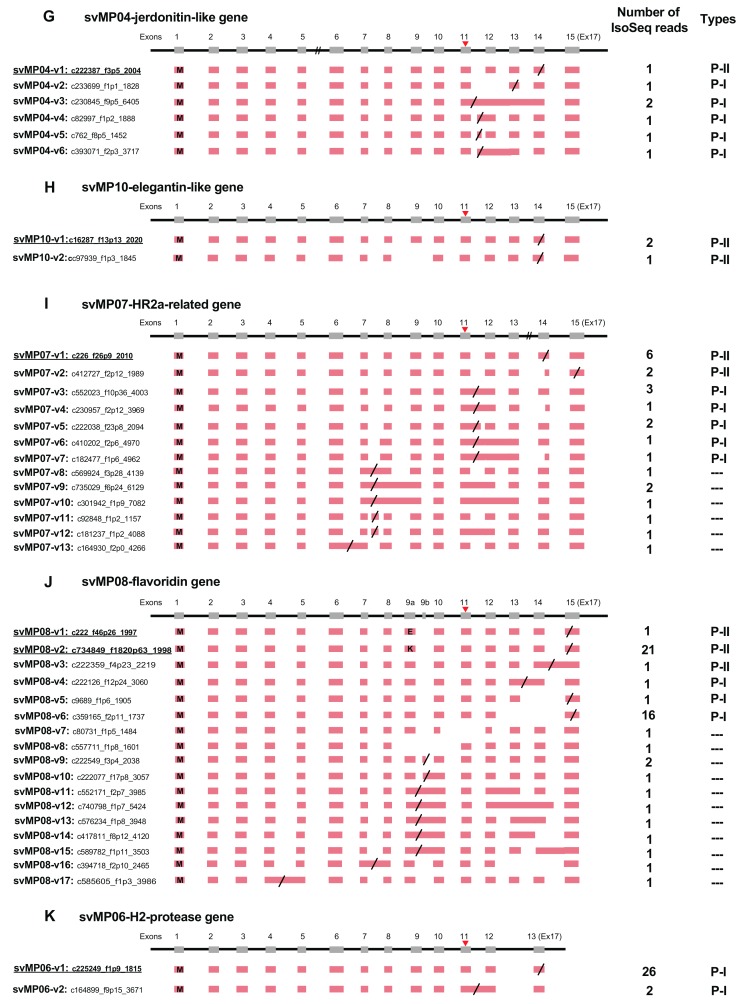
Schematic diagram of structures of metalloproteinase (MP) genes expressed in habu venom glands. *svMP03-VMP-III-like* (**A**), *svMP01-HV1* (**B**), *svMP02-flavorase* (**C**), *svMP09-HR1b-related* (**D**), *svMP05-HR1a-relate* (**E**), *svMP11-NaMP* (**F**), *svMP04-jerdonitin-like* (**G**), *svMP10-elegantin-like* (**H**), *svMP07-HR2a-related* (**I**), *svMP08-flavoviridin* (**J**), and *svMP06-H2-protease* (**K**) are shown with validated transcript variants, verified as expressed in the venom gland. Initiation and stop-codons are marked with “M” and slashes, respectively. Active sites are shown with red arrowheads located in exon 11. The original transcripts predicted by the gene models are underlined. Both svMP08-v1 and v2 are original transcripts, of which amino acid 275 are indicated by E(Glu) and K (Lys), respectively. Numbers of transcript variants and the type of metalloproteinases are shown on the right.

**Figure 2:**
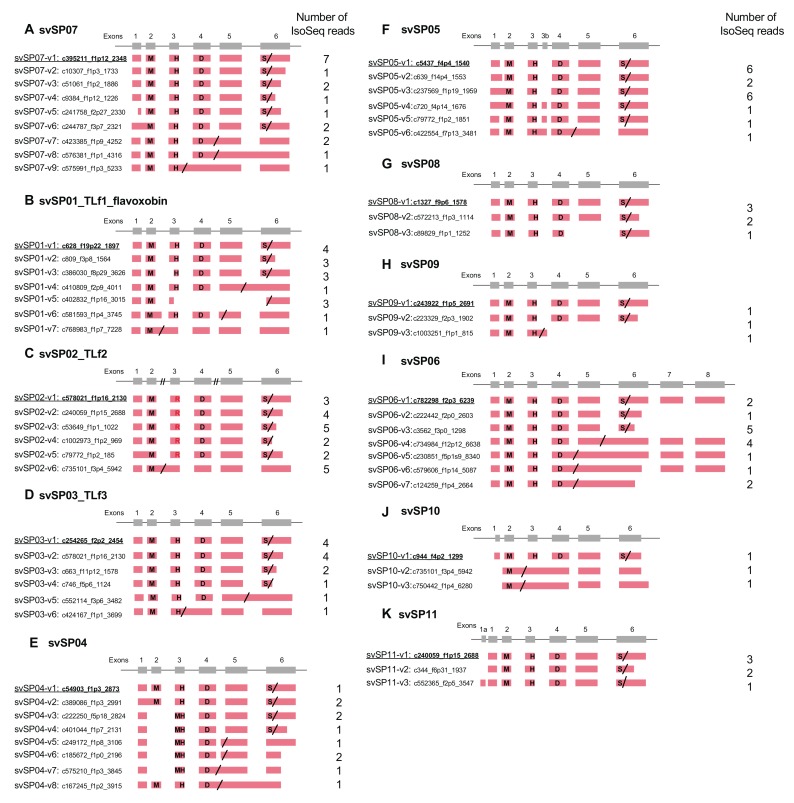
Schematic diagram of structures of serine protease (SP) genes expressed in habu venom glands. *svSP07* (**A**), *svSP01-TLf1-flavoxobin* (**B**), *svSP02-TLf2* (**C**), *svSP03-TLf3* (**D**), *svSP04* (**E**), *svSP05* (**F**), *svSP08* (**G**), *svSP09* (**H**), *svSP06* (**I**), *svSP10* (**J**), and *svSP11* (**K**) are shown with validated transcript variants, verified as expressed in the venom gland. Initiation and stop-codons are marked with “M” and slashes, respectively. His, Asp, and Ser residues of the catalytic triad are indicated as “H”, “D”, and “S”, respectively. In *svSP02-TLf2*, the His residue “H” of the catalytic triad is substituted by Arg (R). Numbers of transcript variants are shown on the right. The original transcripts predicted by the gene models are underlined.

**Figure 3:**
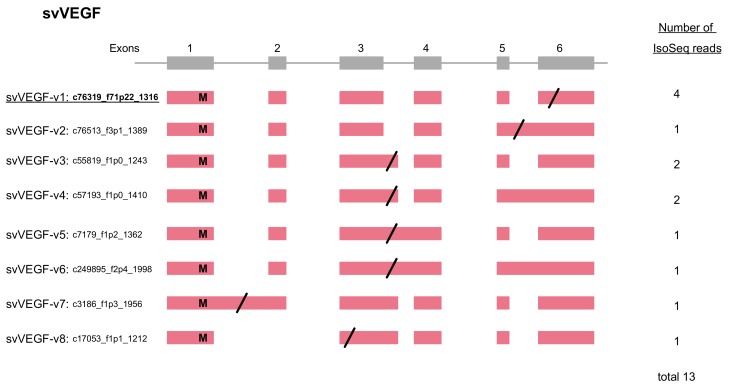
Schematic diagram of structure of vascular endothelial growth factor (VEGF) genes expressed in habu venom glands. Initiation and stop-codons are marked with “M” and slashes, respectively. Seven transcript variants, svVEGF-v1 to v8, were identified. Numbers of transcript variants are shown on the right. The original transcript predicted by the gene models is underlined.

**Figure 4:**
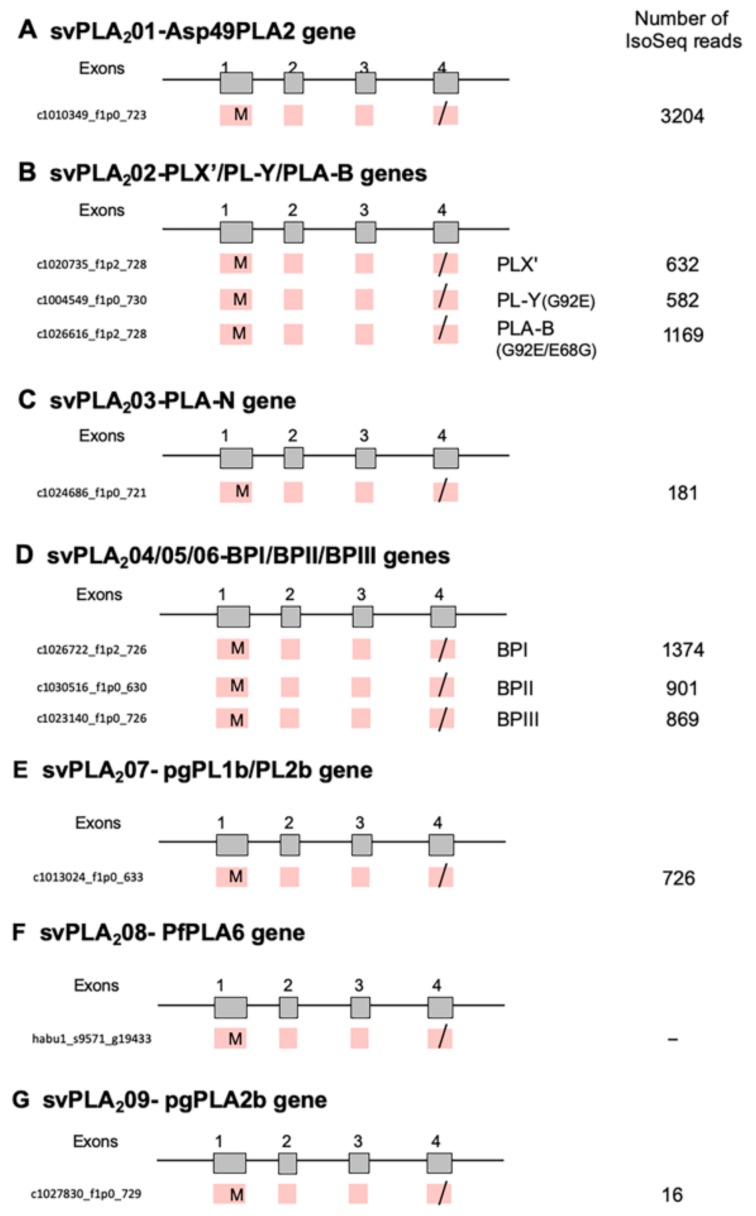
Schematic diagram of structure of phospholipase A_2_ (PLA2) genes expressed in habu venom glands. *svPLA2_01-Asp49PLA2* (**A**), *svPLA2_02-PLX’/PL-Y/PLA-B* (**B**), *svPLA2_03-PLA-N* (**C**), *svPLA2_04/05/06-BPI/BPII/BPIII* (**D**), *svPLA2_07-pgPL1b/PL2b* (**E**), *svPLA2_08-PfPLA6* (**F**), and *svPLA2_09-pgPLA2b* (**G**) are shown with validated transcript variants expressed in the venom gland. Initiation and stop-codons are marked with “M” and slashes, respectively. Numbers of transcript variants are shown on the right.

As indicated, our previous study demonstrated 18 families of 60 venom and 224 non-venom counterpart genes [7]. In the previous study, we also obtained a total 97,405 reads of cDNA collected from the venom gland using PacBio RSII (Iso-Seq) (a total read-length = 179,143,509 bp). We manually annotated these full-length transcripts corresponding to the gene models previously constructed. As a result, we observed alternative splicing exclusively in three families of venom genes: MPs, SPs, and VEGFs (Figure 1, Figure 2 and Figure 3). No other families of venom genes, including PLA2s, showed alternative splicing (Figure 4). In other words, each of the transcripts of the remaining 15 venom gene families showed one-to-one correspondence to appropriate venom gene models. svMP and svSP are two of the four major components of habu venom. In contrast, svVEGF is a member of the group in which a single-copy gene is transcribed into one corresponding mRNA. This indicates that (1) alternative splicing contributes to the production of mRNA variants exclusively in venom genes, and (2) alternative splicing occurs only in a few venom gene families, not commonly shared by all venom gene families.

### 2.2. Alternative Splicing of 10 svMP Genes Produces 81 mRNA Variants

**Figure 5:**
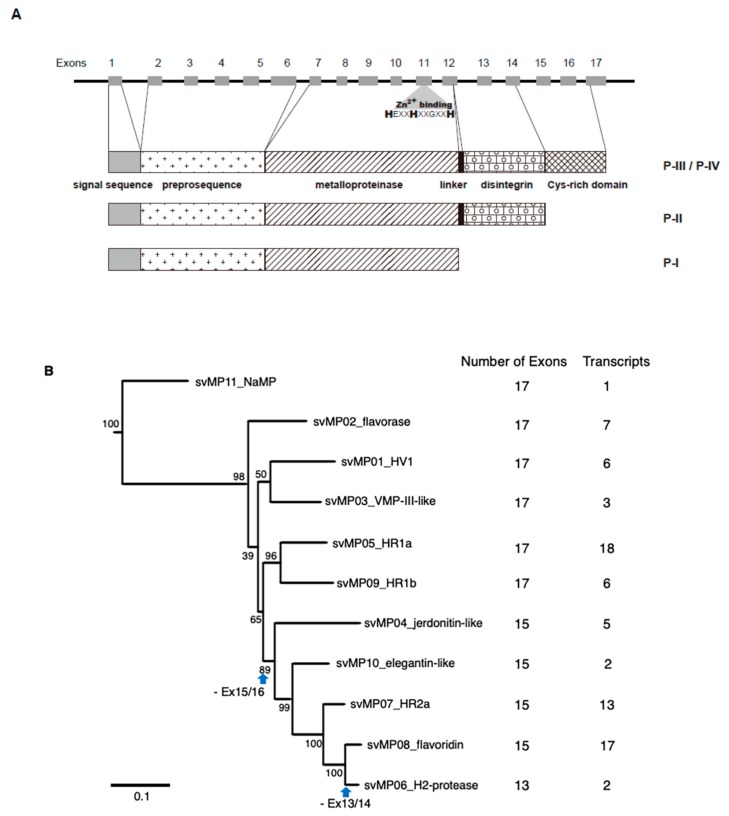
Schematic structures (**A**) and phylogenetic tree (**B**) of metalloproteinase genes expressed in habu venom glands. (**A**) The gene structure is shown with 17 exons. A Zn binding site is also shown in exon 11. Different domains in the three types of MP protein products (P-I, P-II, and P-III/P-IV) are shown as boxes. The active site is also shown by asterisk as “Zn^2+^ binding” motif, HEXGHNLGXXHD. P-IV contains lectin-like domain linked by disulfide bonds to the P-III structures. (**B**) Molecular phylogeny of 11 *MP* genes. Three non-venom MPs found in the habu genome, *nvMP08*, *nvMP09*, and *nvMP11* were used as outgroups for rooting the tree. Bootstrap probabilities of 1000 replications are shown at each node. Deduced timings of gain and loss of exons are shown by blue arrows. Exon structures and numbers of transcript variants are also shown on the right.

MPs are key toxins that cause venom-induced pathogenesis such as hemorrhage, fibrinolysis, and apoptosis. According to their domain architecture, svMPs are classified into four groups (P-I to P-IV) [11] (Figure 5A). P-I MPs possess only the metalloproteinase domains and are largely non-hemorrhagic. P-II MPs contain MP domains and disintegrin domains. P-III MPs contain Cys-rich domains as well as MP and disintegrin domains and exhibit great diversity in structure and function. P-IV MPs harbor lectin-like domains linked by disulfide bonds to the P-III-like structures [11]. Eleven *svMP* genes have been annotated from the habu gene model, HabAm1 (*svMP01* to *svMP11*) (Figure 1 and Figure 5B; Supplementary Table S1).

In the present study, we obtained 1777 PacBio reads related to the svMPs, however only 1032 PacBio reads out of 1777 can be assigned on the *svMP* genes, *svMP01* to *svMP10*. Residual 745 PacBio reads for svMPs could not assigned on *svMP* genes probably due to their non-perfect match to the known svMPs. This suggests that the additional new svMP genes exist in the habu genome. After removing the short PacBio reads (total 839 reads), 193 full-length transcripts (PacBio reads) that include both start Met codon and polyA signal were used for further analysis.

As shown in Figure 1 and Figure 5B, the *svMP* genes consist of 13 to 17 exons. Six *svMP* genes, *svMP01-HV1*, *svMP02-flavorase*, *svMP03-VMP-III-like, svMP05-HR1a-related*, *svMP09-HR1b-related* and *svMP11-NaMP-like* consist of 17 exons. Four *svMP* genes, *svMP04-jerdonitin-like*, *svMP07-HR2a-related*, *svMP08-flavoridin,* and *svMP10-elegantin-like* consist of 15 exons. Only *svMP06-H2-protease* consists of 13 exons. Exon 1 encodes a signal sequence, and exons 2 to 6 encode pre-prosequences. Exons 7 to 12 encode MP domains, including a catalytic zinc binding motif, HEXGHNLGXXHD encoded by exon 11 in all *svMP* genes (Figure 5A). Furthermore, exons 13 and 14 encode the disintegrin domains of P-II and P-III svMPs, and exons 15 and 16 encode the Cys-rich domain of P-III svMPs (Figure 5A). A molecular phylogenetic tree constructed from nucleotide sequences of *P. flavoviridis* svMPs is shown in Figure 5B. The gene structure with 17 exons, as seen in *svMP11-NaMP-like*, is suggested to be ancestral due to its basal location in the phylogenetic tree (Figure 5B)*. svMP02-flavorase, svMP01-HV1, svMP03-VMP-III-like, svMP05-HR1a-related*, and *svMP09-HR1b-related* have diverged from this ancestor without any structural changes to exons. On the other hand, exons 15 and 16 corresponding to the Cys-rich domain were deleted in the four genes consisting of 15 exons: *svMP04-jerdonitin-like, svMP10-elegantin-like, svMP0*7*-HR2-related*, *svMP08-flavoridin*, and *svMP06-H2-protease* (Figure 1 and Figure 5B).

In the case of *svMP06-H2-protease,* exons 13 and 14 corresponding to the disintegrin domain were further deleted, resulting in 13 exons (Figure 5B). An important notion is that the last exon, exon 17 in the original construct, is conserved in all of the “reduced” gene copies consisting of 13 or 15 exons. These gene structures suggest that the ancestral *svMP* gene copy related to *svMP11-NaMP* consisting of 17 exons duplicated into multiple copies, and some of the copies were then modified by two-round deletions, the first one involving loss of exons 15 and 16, and the second one resulting in further loss of exons 13 and 14, resulting in 11 *svMP* gene copies with different numbers of exons (Figure 5B).

All of the 81 full-length mRNAs expressed in the venom gland matched each of the corresponding gene models. We identified multiple kinds of transcripts for each of the MP genes except for *svMP11-NaMP,* which expressed only one type of the transcript (Figure 1F; Supplementary Table S1). We hereafter call the transcript completely matching the corresponding gene model as “original” represented by svMP03-v1 for *svMP03-VMP-III-like* supported by six Iso-Seq reads (Figure 1A). Besides the original transcripts, we observed 2, 5, 6, 5, 18, 0, 5, 1, 12, 16, and 1 variant transcripts for the other *MP* genes. (Figure 1A–K). These variants include transcripts, of which 3′ untranslated regions’ (3′UTR) length is the only thing that is different due to having multiple polyA signals AATAAA, and which are able to translate the same protein products. For example, they include svMP03-v1 and svMP03-v2 for *svMP03-VMP-III-like*, svMP01-v1, svMP01-v2 and svMP01-v3 for *svMP01-HV1*, svMP02-v1, svMP02-v2 and svMP02-v3 for *svMP02-flavorase*, and svMP09-v2 and svMP09-v3 for *svMP09-HR1b*. We also observed variable positions of the stop-codon in *svMP01-HV1-related*, *svMP02-flavorase-related*, *svMP09-HR1b-related*, *svMP05-HR1a-related*, *svMP04-jerdonitin-like*, *svMP07-HR2a-related*, *svMP08-flavoridin,* and *svMP06-H2-protease* (Figure 1B–E,G,I–K). Most of these differences are associated with exon skipping or intron retention followed by frame shifts. For example, three different transcript variants of *svMP09-HR1b* show variable positions of the stop-codon, svMP09-v4 skipping exon 15 with a new stop-codon in exon 16, svMP09-v6 retaining intron 13 with a new stop-codon in the newly fused exon (exons 13 and 14), and svMP09-v5 retaining intron 14 with a new stop-codon in the newly fused exon (exons 14 and 15) (Figure 1D), resulting in the variants corresponding to P-I and P-II MPs, respectively.

The most extensive variations were noted in *svMP05-HR1a-related,* showing the original transcript svMP05-v1 supported by 10 Iso-Seq reads and 17 variant transcripts, svMP05-v2 to svMP05-v18, supported by 1 to 4 Iso-Seq reads, respectively (Figure 1E). Unlike other *svMP* genes, extra sub-exons (1b, 1c, 1d, and 1e) were observed between the original exon 1 and exon 2 in some transcript variants such as svMP05-v2, The original transcript and 12 variants contained only the original exon 1a, while another six transcript variants exhibited more complicated combinations of exon usage characterized by loss or fusion of certain exons (svMP05-v2, svMP05-v3, svMP05-v4, svMP05-v5, and svMP05-v6 in Figure 1E). Five of the six variants also showed different positions of the start-codon due to different exon usage (svMP05-v3, svMP05-v4, svMP05-v5, and svMP05-v6 in Figure 1E). Six exons, exon 7 to exon 12 encoding the MP domain, including the catalytic Zn finger motif (in exon 11), are conserved in all of the observed transcripts, suggesting that the six exons are essential for enzyme function (Figure 1 and Figure 5A). It is notable that the original transcript of *svMP05-HR1a-like* encodes P-III MP, while two other types of svMPs are also transcribed from the same gene by alternative splicing. Six transcripts encode P-I type, in which a stop-codon was introduced after exon 12 due to the retention of intron 12 (transcripts svMP05-v12, svMP05-v14, svMP05-v15, and svMP05-v16). Another two transcripts encode P-II-type in which a stop-codon was introduced after exon 14 due to the retention of intron 14 (transcript: svMP05-v9) or shortening of exon 14 (transcript: svMP05-v10).

*svMP04-jerdonitin-like, svMP10-elegantin-like, svMP0*7*-HR2a-related*, and *svMP08-flavoridin* are likely to comprise another group of *svMP* genes of which the original construct is composed of 15 exons encoding P-II MPs (Figure 1G–J). As observed above for the genes that originally consisted of 17 exons, variable transcripts were produced by exon skipping and intron retention (Figure 1G–J). The most extensive variation in this group was observed in *svMP08-flavoridin,* which is expressed as 15 variant transcripts and two original ones (Figure 1J). Both svMP08-v1 and svMP08-v2 were original transcripts, of which amino acid 275 is replaced by Glu or Lys. Another transcript, svMP08-v3, carrying fused exons 14 and 15 by retaining intron 14 was a P-II type with a disintegrin domain. However, three variant transcripts, svMP08-v4 carrying fused exons 13 and 14 by retaining intron 13, svMP08-v5 eliminating exon 14 and svMP08-v6 eliminating exons 14 and 15, were P-I type. Extensive variations were also observed in *svMP07-HR2a-related,* expressed as 12 variant transcripts, svMP07-v2 to v13 as well as the original transcript (Figure 1I). Although the first five exons, exon 1 to 5, are shared by all of the nine transcripts, intron retentions are involved in the variant transcripts, svMP07-v3 carrying fused exons 11 and 12 by retaining intron 11, svMP07-v2 carrying short exon 14, c110395_f1p2_2139 carrying extended exon 8, svMP07-v5 carrying extended exon 11 by partial retain of intron 11, svMP07-v13 retaining intron 6, svMP07-v8 retaining intron 7, svMP07-v9 retaining introns 7 and 11, and svMP07-v11 and svMP07-v12 partially retaining intron 7 (Figure 1I). New stop-codons are gained in the retained intron 11 in two transcripts encoding P-I MPs: svMP07-v3 and svMP07-v5, which can be produce the same protein. Lastly, *svMP06-H2-protease,* consisting of 13 exons, is transcribed into a variant, svMP06-v2 gaining a stop-codon in the retained intron 11 as well as the original construct (Figure 1J).

**Figure 6:**
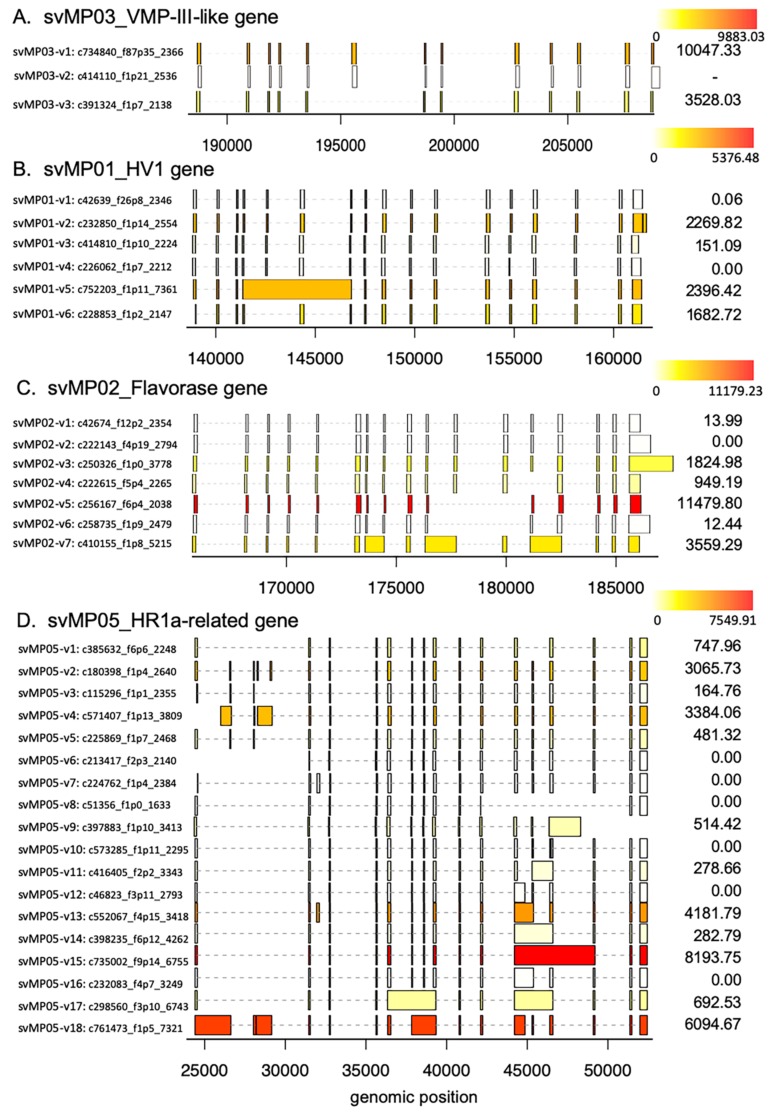

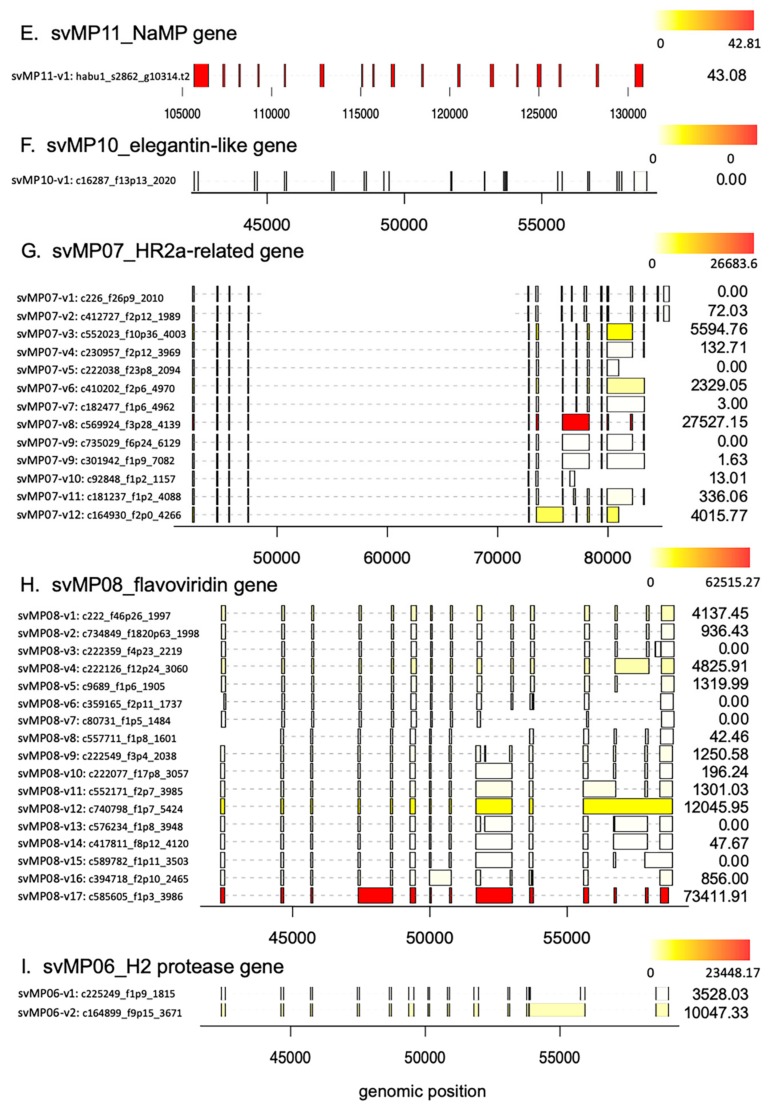
Structure and expression levels of distinct transvariant forms of *svMPs* genes in habu venom glands. (**A**) *scMP03_VMP-III like*, (**B**) *svMP01_HV1*, (**C**) *svMP02_flavorase*, (**D**) *svMP05_HR1a*, (**E**) *svMP11_NaMP*, (**F**) *svMP10_elegantin-like*, (**G**) *svMP07_HR2a*, (**H**) *svMP08_flavoviridin*, and (**I**) *svMP06_H2 protease*. Expression levels are shown on the right with FPKM values.

To confirm the expression level of each transcript variant for svMPs, we analyzed the prevalence and relative abundance of the splice variants in venom gland by HISAT (hierarchical indexing for spliced alignment of transcripts), StringTie, and Ballgown with Illumina RNA-Seq data (Figure 6). The relative abundance of the splice variants for other svMPs were obtained except for svMP04 and svMP09. They could not be obtained due to their gene structures locating across the multi-scaffolds, that is, svMP04 is located on habu1_scaffold415864, habu1_scaffold191139, and habu1_scaffold403873, and svMP09 is located on habu1_scaffold3258, habu1_scaffold399953, habu1_scaffold411246, habu1_scaffold399953, and habu1_scaffold400013. These data suggest the existence of the several splice variants of svMPs in venom gland. For example, in the case for *svMP05-HR1a* gene, distinct expression of transcript variants for svMP05-v1 to v18 was detected by Illumina RNA-seq except for svMP05-v6, svMP05-v7, svMP05-v8, svMP05-v10, svMP05-v12, and svMP05-v16. These differences of expression profiles between Illumina RNA-seq and PacBio reads may be individual specificities. Interestingly, svMP05-v15 corresponding to P-I type MP showed higher expression (Fragments Per Kilobase of exon per Million mapped fragments (FPKM) 2769.20) compare with svMP05-v1 corresponding to P-III type MP, HR1a (FPKM 247.38) (Figure 6D).

As described, svMPs are classified into four different groups (P-I to P-IV). Different types of MP proteins may be produced from single *MP* genes such as *svMP09-HR1b-related* and *svMP05-HR1a-related* not only by proteolytic processing but also alternatively splicing, resulting in a variety of svMPs and disintegrin peptides.

### 2.3. Alternative Splicing of svSP Genes Produces mRNA Variants with Different Lengths of 3′UTR or Inactive Short Forms Lacking C-Terminal Region

SPs are another major group of protein components of snake venom. We identified 11 habu *svSP* genes (*svSP01* to *svSP11*) from the gene model of HabAm1 (Figure 2; Supplementary Table S2) [7]. *svSP01* is known as flavoxobin, TLf1, or habutobin, which is the most abundantly expressed *svSP* in the venom gland [12,13,14]. It encodes a thrombin-like enzyme of 242 amino acids that specifically releases fibrinopeptide A from fibrinogen [12]. *svSP02* corresponds to another major svSP transcript encoding a basic thrombin-like enzyme, TLf2. Comparative analyses of nucleotide sequences of *P. flavoviridis* svSPs with orthologues previously reported from other snake species [15] enabled us to assign *svSP03, svSP04, svSP05*, and *svSP09* genes to basic thrombin-like enzyme TLf3, a new SP homologous to stejnefibrase-1, flavorase, and an α-fibrinogenase, respectively.

**Figure 7:**
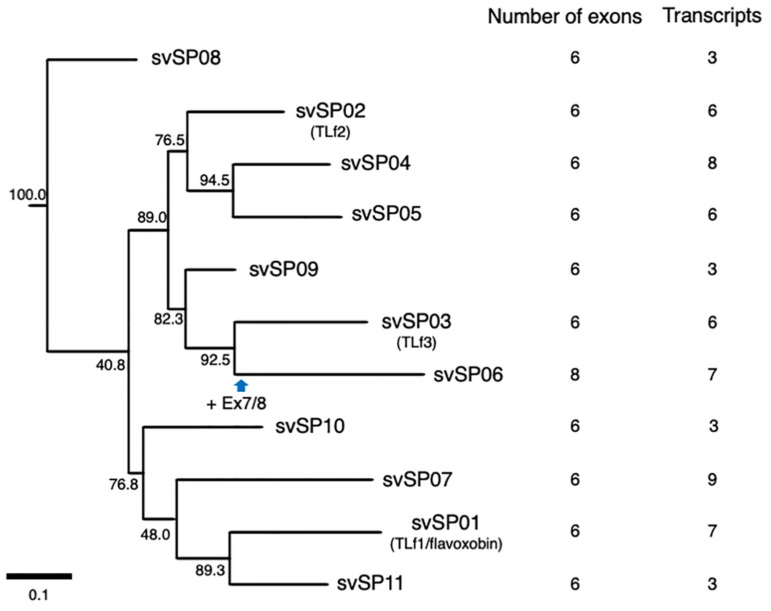
Molecular phylogeny of serine protease genes expressed in habu venom glands. A non-venom SP found in the habu genome, *nvSP05-kallikrein*, was used as an outgroup for rooting the tree. Bootstrap probabilities of 1000 replications are shown at each node. Deduced timing of gain of exons is shown by a blue arrow.

All *svSP* genes are composed of six exons, except *svSP06* which has two new exons (Figure 2 and Figure 7). A molecular phylogenetic tree of the 11 *svSP* genes suggests that *svSP08* is likely to be the earliest-diverged variant from the ancestral state, followed by multiple stages of duplications and divergence (Figure 2). As shown in Figure 2 and Figure 7, in all 11 *svSP* genes, irrespective of the number of exons, the first exons correspond to 5′ UTRs. All of these *svSPs* share the same exon–intron junctions as the coding regions of mammalian kallikrein (*KLK*) genes [16]. Together with the similarity of enzymatic activities and the conservation of primary and tertiary structures of SPs, it has been thought that the ancestral copy of all *svSPs* is a kallikrein-like SP rather than other types of SPs such as elastase. This further suggests that *svSP06* gained two new exons, exons 7 and 8, resulting in extension of the 3′ UTR (Figure 2I).

**Figure 8:**
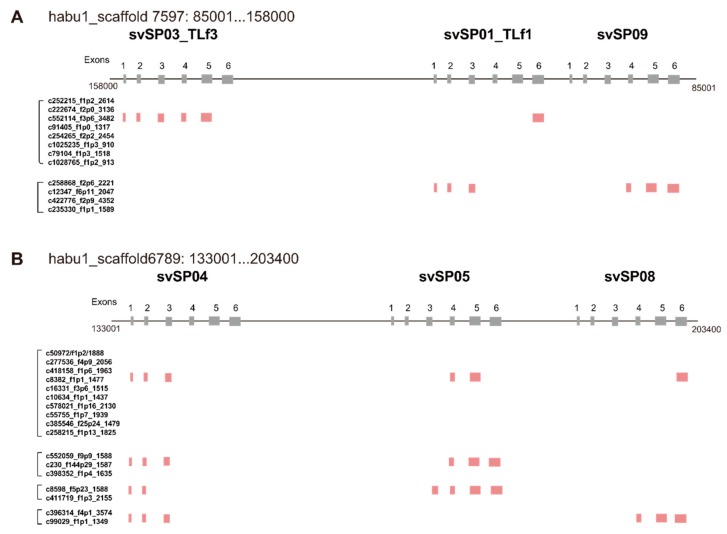
Chimeric transcripts generated by trans-splicing of clustered genes. Trans-spliced transcripts were identified from two regions of *SP* gene clusters, habu1_scaffold 7597: 85001...158000 (**A**) and habu1_scaffold6789: 133001...203400 (**B**). All of these trans-spliced transcripts were supported by multiple Iso-Seq reads.

A great variety of transcripts of *svSP* genes was observed, indicating extensive alternative splicing of *svSP* genes as seen in *svMP* genes (Figure 2 and Figure 7). *svSP08*, *svSP09*, *svSP10* and *svSP11* express three transcript variants each, including the “original” transcript deduced from the gene model. *svSP02-TLf2*, *svSP03-TLf3*, and *svSP05* are transcribed into six variants. *svSP01-TLf1-flavoxobin* and *svSP06* are transcribed into seven variants. *svSP04* is transcribed into eight variants. *svSP07* is transcribed into nine variants. As seen in MP genes, we noticed several mechanisms to produce transcript variants. For example, among the eight transcripts expressed from *svSP04*, skipping of exon 2 was observed in five transcript variants, resulting in gain of a new start-codon in exon 3 of the original construct (svSP07-v3, svSP07-v4, svSP07-v5, svSP07-v6, svSP07-v7) (Figure 2E). Furthermore, variant transcripts completely or partially retaining intron 4 result in gaining a new stop-codon in the original intron 4 (svSP07-v5, svSP07-v6, svSP07-v7, and svSP07-v8). In addition, unlike *svMP* genes, the svSP transcript variants show variable lengths in the 3′-region of exon 6 due to multiple polyA signals (Figure 2A–D,G,H,K). Furthermore, we observed variant transcripts derived from trans-splicing involving exons in two to three tandemly arranged neighboring genes (Figure 8). For example, c252215_f1p2_2614 was transcribed from exons 1–5 of *svSP03-TLf3* and exon 6 of *svSP01-TLf1-flavoxobin* which is located downstream of *svSP03-TLf3* (Figure 8A). c258868_f2p6_2221 was transcribed from exons 1–3 of *svSP01-TLf1-flavoxobin* and exons 4–6 of *svSP09* which is located downstream of *svSP01-TLf1-flavoxobin* (Figure 8A). c50972_f1p2_1888 was transcribed from exons 1–3 of *svSP04*, exons 4 and 5 of *svSP05* and exon 6 of *svSP08*. c552059_f9p9_1588 was transcribed from exons 1–3 of *svSP04* and exons 4 and 5 of *svSP05* (Figure 8B). c8598_f5p23_1588 was transcribed from exons 1 and 2 of *svSP04* and exons 3–6 of *svSP05* s (Figure 8B). c39314_f4p1_3574 was transcribed from exons 1–3 of *svSP04* and exons 4–6 of *svSP08* genes (Figure 8B). All of these trans-spliced products were supported by multiple Iso-Seq reads. Therefore, intrinsic trans-splicing serves as a previously unknown mechanism to enhance the diversity of final protein products of *svSP* genes.

### 2.4. VEGF Gains Its C-Terminal Modification by Alternative Splicing

VEGFs are key regulators of vascular development during embryogenesis (vasculogenesis), blood-vessel formation (angiogenesis), skeletal growth, and reproductive functions [17]. We identified three *VEGF* genes from the gene model of HabAm1. One is the toxic copy, *svVEGF01* (habu1_s565_g02679) encoding svVEGF-F, while the other two are non-toxic copies, *nvVEGF01* (nv: non-venom; habu1_s6836_g17529) and *nvVEGF02* (habu1_s9381_g19343) encoding VEGF-A and VEGF-C, respectively. In the current analysis we identified eight transcript variants expressed from *svVEGF01* (Figure 3). The “original” transcript, svVEGF-v1 was the major transcript encoding the full-length length amino acid sequence exactly matching the one previously reported [18]. In contrast, all transcript variants carry variably shortened coding sequences. svVEGF-v2 gained a stop-codon in the retained intron 5. In spite of the difference in structures of exons 5 and 6, svVEGF-v3, svVEGF-v4, svVEGF-v5, and svVEGF-v6 commonly gained a stop-codon in the retained intron 3, resulting in an identical protein product. svVEGF-v7 gained a stop-codon in the retained intron 1, resulting in a highly truncated protein. svVEGF-v8 gained a new stop-codon in exon 3 due to skipping of exon 2, resulting in a very short protein.

### 2.5. PLA_2_s Show No Alternative Splicing

PLA_2_ (EC 3.1.1.4) catalyzes the hydrolysis of 2-acyl ester bonds of 3-*sn*-phosphoglycerides in the presence of Ca^2+^, to liberate 3-*sn*-lysophosphoglycerides and fatty acids. Habu snake genome contains nine types of group II venom *PLA_2_* genes (*svPLA_2_01* to *svPLA_2_09*) (Figure 4) [7], including a hemolytic [Asp49]PLA_2_ (*svPLA_2_01*), edema-inducing basic [Asp49]PLA_2_ including PLX’, PL-Y and PL-B (*svPLA_2_02*), a weak neurotoxin PLA-N (*svPLA_2_03*), and [Lys49]PLA_2_ myotoxins (*svPLA_2_04* to *svPLA_2_06* corresponding to BPI, BPII, and BPIII, respectively), were expressed in the venom gland with high number of transcripts (16–3206) compared with three genes, *svMP*, *svSPs*, and *svVEGF* (Figure 4). From the structure of each transcript, svPLA_2_s have no alternative splicing although these *PLA_2_* genes consist of four exons (Figure 4).

Total number of transcripts for PLA_2_s was estimated to be 9654, ten percent of 97,405 Iso-Seq reads in venom gland. Compared with PLA_2_s, total number of reads for svMP, svSPs, and svVEGF were low with 1777, 476, and 20, respectively. On the other hand, FPKM values of svPLA_2_s, svMPs, svSPs, and svVEGF in venom glands from two individuals (VG1/VG2) were estimated to be 3590.15/13728.43, 6140.1/1138.4, 149.2/630.8, and 90.4/116.9, respectively. These results suggest that the expression levels of svPLA_2_s, svMPs, svSPs, and svVEGF estimated by two different methods, PacBio Iso-Seq reads and Illumina RNA-seq, were almost parallel. However, the number of full-length transcripts (Iso-Seq reads) for svMPs were very low, more than the difference of expression levels probably due to their short reads and the existence of unidentified *svMP* genes. Furthermore, PLA2s are much easier to identify (only four exons, <450 bp usually for the coding sequence) so the models may be much better for them. With svMPs having 15–17 exons (>1400 for Type 2 and >1800 for Type 3), there is a lot more possibility of errors in annotation.

## 3. Discussion

Previous studies have suggested the occurrence of alternative splicing of venomic *SP* genes in *Vipera lebetina* and *Bitis gabonica rhinoceros* [9,10]. Recent high-throughput transcriptomics (RNA-seq) demonstrated a highly divergent profile of mRNA species encoded by venom genes, and the involvement of alternative splicing is suggested to contribute to transcriptome variation [4,5,6]. Genome-wide transcriptome analyses, including alternative-splicing variants of venom genes, have not been previously reported. By genome-based comprehensive analysis of the full-length transcript information provided by Iso-Seq reads of the venom gland in *P. flavoviridis*, we showed that only three of the 18 families, namely *svMP*, *svSP*, and *svVEGF*, produce highly diverse transcript variants by alternative-splicing in venom glands. Thus, the diversity of venom transcripts in *P. flavoviridis* is provided by two distinct mechanisms; namely, alternative splicing, as we reported in the current study, and the increment of copy number followed by accelerated evolution, as we reported earlier [7].

The present study disclosed “double-decker” mechanisms; namely, extensive gene duplication followed by the rearrangements of exon/intron structures. For examples, in the case of the 24 copies of *svMP* genes, five copies preserved the original arrangement consisting of 17 exons, whereas four copies are the “deleted” construct consisting of 15 exons, of which the terminal one is “further deleted” carrying only 13 exons. The most extensive variation by alternative splicing was noticed in svMPs; that is, transcript variants involving exon skipping or intron retention (fused exons) incorporated a new stop-codon, resulting in the production of diverse variants corresponding to different types of MPs. For example, from the genes encoding P-III-type MPs, HR1a, and HR1b, all three types of variants were produced, P-I, P-II, and P-III. While from the P-II-type MP genes such as *svMP04-jerdonitin-like*, *svMP07-HR2a-related*, and *svMP08-flavoridin*, two types variants were produced, P-I and P-II. These transcript variants for each MP were confirmed by HISAT, StringTie, and Ballgown with Illumina RNA-Seq data (Figure 6). The diversification mechanism by alternative splicing may be advantageous for svMPs because they are mosaic proteins belonging to a disintegrin and metalloprotease (ADAM) family, which contains the disintegrin and Cys-rich domains encoded by separate exons, in addition to MP and pre-prosequence domains (Figure 5A).

In the case of the 11 copies of *svSP* genes, 10 copies retained the original architecture consisting of six exons, whereas one copy gained two new exons, resulting in eight exons. Many of these rearrangements involve gain and loss of start- and stop-codons. We identified transcript variants for all of the 11 *svSP* genes carrying intron retentions as well as exon skipping, resulting in gains of premature stop-codons or of secondary start-codons. Some transcript variants such as c768983_f1p7_7228 of *svSP01-TLf1-flavoxobin* and c735101_f3p4_5942 of *svSP02-TLf2* are very small and naturally devoid of catalytic activity as proteinases due to lack of the catalytic residue, Ser195. Our unpublished data show that TLf2 enhances the myonecrotic activity of [Lys49]PLA_2_, although TLf2 was hydrolytically inactive due to replacement of its catalytic residue His57 with Arg (Ogawa et al., in preparation). This indicates the possible functions of svSP fragmentary products derived from short-transcript variants because TLf2 exhibits a unique activity independent of its protease activity.

In addition to the two major protein components in the venom MP and SP, alternative splicing also serves to enhance the diversity in transcripts expressed from *svVEGF* (Figure 3). Transcript variants of *svVEGF* carry variable stop-codon positions, such as retained intron 1, exon 3, retained intron 3, and retained intron 5 and exon 6 (original). VEGFs in snake venoms are known to show highly strict specificities of receptor-binding compared to mammalian VEGFs. For example, vammin and VR-1, isolated from the venoms of *Vipera ammodytes* and *Daboia russelii*, respectively, bind only kinase insert domain receptor (KDR) with high affinity but not to other VEGF receptors [19]. It is known that the C-terminal regions of VEGFs are one of the determinants for receptor specificities [20,21]. Yamazaki et al. [18] has also reported that svVEGF of *P. flavoviridis* preferentially binds to Flt-1 rather than to KDR, unlike vammin and VR-1 [22]. Thus, transcript variants of svVEGFs with variable C-terminal regions are likely to show differences in receptor specificities, rendering additional diversity in physiological functions of the venom.

Besides the normal cis-spliced transcripts discussed above, we also observed trans-spliced transcript variants of *svSPs* expressed from genes tandemly clustered so that the pre-mRNA spanning multiple genes is able to be expressed (Figure 8). This may serve as yet another mechanism, which is previously unknown, to enhance the diversity of venom protein products. In the current study, we reported trans-splicing involving two regions of clustered genes, namely *svSP03-svSP01-svSP09* and *svSP04-svSP05-svSP08*. In our previous study, however, we identified more cases of gene clusters of *MPs*, *SPs*, and *CTLPs* in the habu genome. Therefore, it is highly likely that trans-splicing also contributes to the expression of other neighboring genes located in the gene cluster, which needs to be elucidated in future study.

Because recent transcriptomic and proteomic analyses of snake venoms have revealed that the venom components often show variations within as well as between species, it is considered that diversification of these venomous proteins at the transcriptional level is advantageous for physiological and environmental changes, including prey species [23,24,25,26,27]. These venom variations may be correlated with geographic location, diverse environments, sexual dimorphism, and feeding behaviors. In the current study, we revealed multilayered mechanisms that enhance the diversity of venom protein products, namely, alternative splicing, and trans-splicing. These are likely to serve as intrinsic mechanisms to render physiological and adaptive changes to the protein components of snake venoms.

## 4. Conclusions

In the present study, we found that alternative splicing was observed in three venom protein gene families, *MP*, *SP*, and *VEGF*, that is, a total of 81, 61, and 8 transcript variants, respectively. Especially, individual genes of *MPs* are transcribed into different classes of MPs by alternative splicing. We also observed trans-splicing among the clustered *SP* genes. No other venom genes such as *PLA2s* exhibited alternative splicing. Thus, our results indicate a significant contribution of mRNA alternative and trans-splicing in the production of highly variable transcripts of venom genes in the habu snake.

## 5. Materials and Methods

### 5.1. Biological Materials

*P. flavoviridis* individuals were collected from Amami-Oshima Island, Kagoshima, Japan in 2011. One female individual was used for RNA extraction from venom gland. Crude venom was collected from adult *P. flavoviridis* from Amami-Oshima, Japan, and lyophilized. It was stored at −30 °C until use.

### 5.2. Transcriptome Analyses by PacBio Reads

Transcriptome analyses by PacBio reads were conducted as described in the previous our work [7]. Briefly, cDNA libraries from the venom gland for PacBio sequencing were prepared by using the manufacturer’s protocol with a SMARTer Pico PCR cDNA Synthesis Kit (TAKARA Clontech) and SMRTbell Template Preparation Kit 1.0 (PacBio). Longer cDNAs were enriched with a SageELF system (Sage Science, Inc.). Sequencing was performed on a PacBio RS II, yielding a total of 179,143,509 bps of 2300 bp average read length. N50 of PacBio reads was 5000 and not required for further assembly. Most of these reads are long enough to be full-length transcripts and directly annotated with BLASTX against UniProt. PacBio reads are publicly available from DDBJ under accession no. DRA006601.

### 5.3. Transcriptome Analyses by Illumina

For transcriptome analyses by Illumina RNA-Seq, two individual venom glands (VG1 and VG2) were used. Total RNA was extracted by using a standard TRIzol protocol procedure (Thermo Fisher Scientific), and cDNA libraries were prepared using an NEBNext^®^ Ultra™ Directional RNA Library Prep Kit for Illumina (New England Biolabs). RNA quality was checked with an Agilent Technologies 2100 Bioanalyzer using an Agilent RNA 6000 Nano Kit. Sequencing was performed using an Illumina Hiseq2500. The sequencing quality was checked by FastQC [28], and low complexity sequences were removed by PRINSEQ [29]. The raw Illumina reads were quality filtered (Q20) and trimmed out 5–10 bp on both ends of the reads to remove bias and low-quality bases by using Trimmomatic [30]. De novo assembly of whole RNA sequence reads was performed using a de Bruijn graph-based program, Trinity [31]. Assembled transcripts were annotated with BLASTX against UniProt. Illumina reads are publicly available from DDBJ under accession no. DRA006600.

### 5.4. Mapping of Transcriptome Data on Genome Sequence and Detection of Transcript Variants

All PacBio Iso-Seq transcript reads were mapped against the genome assembly, HabAm1 using BLAT [32] and PASA [33]. HabuAm1, the genome sequence data of *P. flavoviridis* previously reported [7], can be accessed in BioSamples SAMD00115727 (DRX006596-DRA006599). Accession numbers for scaffolds are BFFQ01000001–BFFQ01084502 (84,502 entries).

PacBio Iso-Seq transcript data encoding venom proteins was checked and corrected via a habu genome browser (Supplementary Figure S1). PacBio Iso-Seq transcript data for venom proteins was also analyzed by blast search on habu1_venom pac bio_prot and habu1_venom_pac_bio_nucl database of the habu genome browser using amino acid sequence and nucleotide sequence of each transcript variant as query sequence. Finally, we confirmed the data by multiple alignments of transcripts against each venom gene with ClustalW [34] and manual curation by comparing with genome sequence to remove the sequencing errors and/or allelic variation. Short Iso-Seq reads without a start Met codon and/or polyA signal were also omitted from data. Then, the number of validated transcript variants were counted.

### 5.5. Bioinfomatic analysis

A genome browser was prepared using the assembled genome sequences using the JavaScript-based Genome Browser (JBrowse) 1.11.6 [35]. The assembled sequence and gene models can be found at http://marinegenomics.oist.jp/habu/.

Alignment of amino acid and nucleotide sequences of each protein family was first performed using ClustalW online at http://clustalw.ddbj.nig.ac.jp. Then the alignment of nucleotide sequences was made based upon the amino acid sequence alignment. We carefully and repeatedly confirmed the correspondence between nucleotide sequences of the genome and mRNAs and amino acid sequences of the predicted proteins.

Phylogenetic trees were constructed by the maximum-likelihood method using IQ-TREE (http://www.iqtree.org) [36], based on aligned nucleotide sequences. Numbers on branches are bootstrap values with 1000 × resampling. The optimal evolutionary model for each phylogenetic tree was selected using ModelFinder [37] implemented in IQ-TREE.

The expression level of transcript variants in venom gland was analyzed by Pertea’s protocols using open source software tools [38], HISAT (hierarchical indexing for spliced alignment of transcripts. Ver. 2.1.0) [39], StringTie (Ver. 1.3.4d) [40], and Ballgown (Ver. 2.16.0) [41] with Illumina RNA-Seq data.

## Supplementary Materials

The following are available online at https://www.mdpi.com/2072-6651/11/10/581/s1, Figure S1: Habu genome browser, Table S1: Metalloproteinase (MP) genes and representative transcripts expressed in the habu venom gland, Table S2: Serine protease (SP) genes and representative transcripts expressed in the habu venom gland.
