# Supplementary material for: Alternative mRNA Splicing in Three Venom Families Underlying a Possible Production of Divergent Venom Proteins of the Habu Snake, Protobothrops flavoviridis
Source: Toxins (Basel). 2019 Oct 9;11(10):581. doi: 10.3390/toxins11100581 (PMC6832531; doi:10.3390/toxins11100581)
Supplement: Supplementary file 1 [file toxins-11-00581-s001.pdf]

Supplementary Materials: Alternative mRNA Splicing in Three Venom Families Underlying a Possible Production of Divergent Venom Proteins of the Habu Snake, *Protobothrops flavoviridis*

Tomohisa Ogawa, Naoko Oda-Ueda, Kanako Hisata, Hitomi Nakamura, Takahito Chijiwa, Shousaku Hattori, Akiko Isomoto, Haruki Yugeta, Shinichi Yamasaki, Yasuyuki Fukumaki, Motonori Ohno, Noriyuki Satoh and Hiroki Shibata

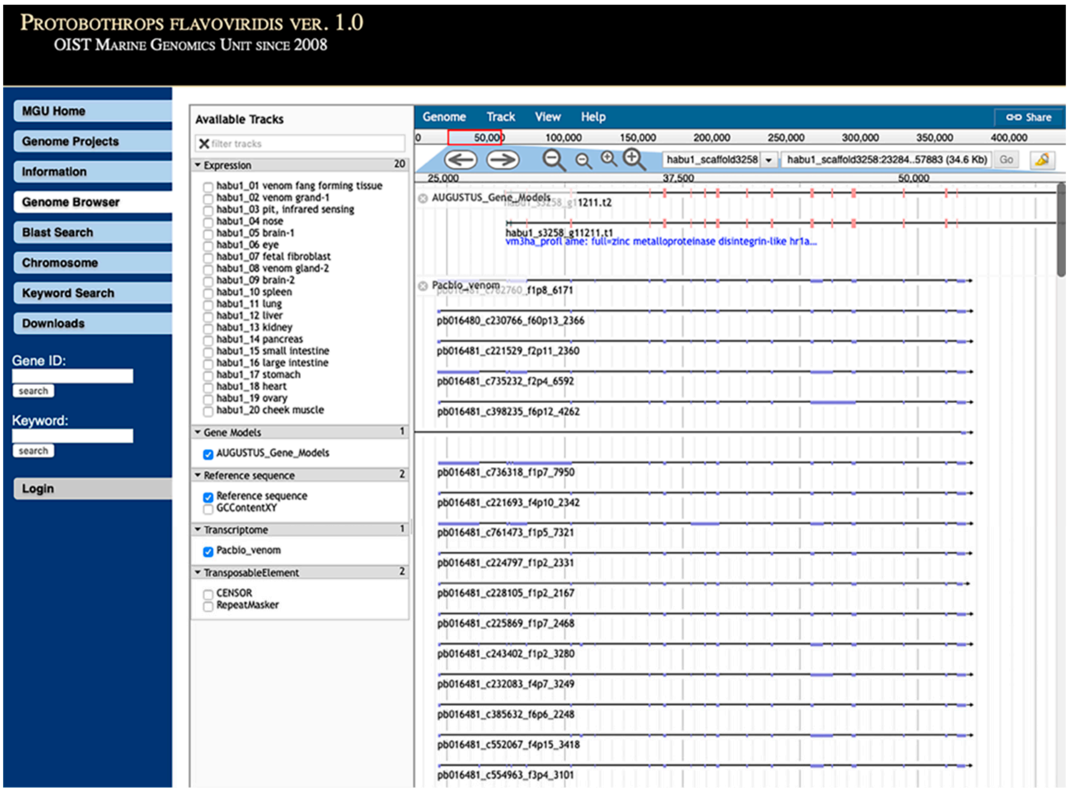

Figure S1. Representative example of Augustus Gene model, *svMP05_HR1a*, with partial Pacbio transcript data.

Table S1. Metalloproteinase (MP) genes and representative transcripts expressed in the habu venom glands.

Gene	Name	Gene Model ID	Transcripts (IsoSeq Reads)	No. of Transcript Valiants in Venom Gland
svMP01	HV1, Vascular apoptosis-inducing protein	habu1_s2862_g10314a	pb016480_c42639_f26p8_2346<br>pb016481_c232850_f1p14_2554<br>pb016481_c414810_f1p10_2224	6

			pb016481_c226062_f1p7_2212 pb016481_c752203_f1p11_7361 pb016481_c228853_f1p2_2147	
svMP02	flavorase	habu1_s2862_g10314b	pb016480_c42674_f12p2_2354 pb016481_c222143_f4p19_2794 pb016480_c250326_f1p0_3778 pb016481_c222615_f5p4_2265 pb016481_c256167_f6p4_2038 pb016481_c258735_f1p9_2479 pb016481_c410155_f1p8_5215	7
svMP03	VMP-III -like	habu1_s2862_g10314c	pb016481_c734840_f87p35_2366 pb016481_c414110_f1p21_2536 pb016481_c391324_f1p7_2138	3
svMP04	jerdonitin-like	habu1_s3258_g11210	pb016481_c222387_f3p5_2004 pb016480_c233699_f1p1_1828 pb016480_c230845_f9p5_6405 pb016480_c82997_f1p2_1888 pb016480_c762_f8p5_1452 pb016481_c393071_f2p3_3717	6
svMP05	HR1a	habu1_s3258_g11211	pb016480_c385632_f6p6_2248 pb016480_c180398_f1p4_2640 pb016480_c115296_f1p1_2355 pb016480_c571407_f1p13_3809 pb016481_c225869_f1p7_2468 pb016481_c213417_f2p3_2140	18

			pb016481_c224762_f1p4_2384<br>pb016480_c51356_f1p0_1633<br>pb016481_c397883_f1p10_3413<br>pb016481_c573285_f1p11_2295<br>pb016481_c416405_f2p2_3343<br>pb016480_c46823_f3p11_2793<br>pb016481_c552067_f4p15_3418<br>pb016481_c398235_f6p12_4262<br>pb016481_c735002_f9p14_6755<br>pb016481_c232083_f4p7_3249<br>pb016480_c298560_f3p10_6743<br>pb016481_c761473_f1p5_7321	
svMP06	H2 metalloproteinase (metalloprotease P-Iia)	habu1_s14911_g21429	pb016481_c225249_f1p9_1815<br>pb016480_c164899_f9p15_3671	2
svMP07	HR2a	habu1_s14911_g21430a	pb016480_c226_f26p9_2010<br>pb016481_c412727_f2p12_1989<br>pb016481_c552023_f10p36_4003<br>pb016480_c230957_f2p12_3969<br>pb016481_c222038_f23p8_2094<br>pb016481_c410202_f2p6_4970<br>pb016480_c182477_f1p6_4962<br>pb016481_c569924_f3p28_4139<br>pb016481_c735029_f6p24_6129<br>pb016480_c301942_f1p9_7082	13

			pb016480_c92848_f1p2_1157 pb016480_c181237_f1p2_4088 /pb016480_c164930_f2p0_4266	
svMP08	flavoridin	habu1_s14911_g21430b	pb016480_c222_f46p26_1997 pb016481_c734849_f1820p63_1998 pb016481_c222359_f4p23_2219 pb016481_c222126_f12p24_3060 pb016480_c9689_f1p6_1905 pb016480_c359165_f2p11_1737 pb016481_c80731_f1p5_1484 pb016481_c557711_f1p8_1601 pb016481_c222549_f3p4_2038 pb016481_c222077_f17p8_3057 pb016481_c552171_f2p7_3985 pb016481_c740798_f1p7_5424 pb016481_c576234_f1p8_3948 pb016481_c417811_f8p12_4120 pb016481_c589782_f1p11_3503 pb016481_c394718_f2p10_2465 pb016481_c585605_f1p3_3986	17
svMP09	HR1b	habu1_s399953_g24864a	pb016481_c917376_f5p9_2037 pb016481_c228750_f1p2_2017 pb016481_c415369_f1p4_2324 pb016481_c222397_f4p4_2395 pb016481_c568766_f1p6_4	6

			063 pb016481_c559926_f1p7_4374	
svMP10	Mt-b/ elegantin-like	habu1_s399953_g24864b	pb016481_c16287_f13p13_2020 pb016480_c97939_f1p3_1845	2
svMP11	NaMP	habu1_s2862_g10314d	habu1_s2862_g10314.t2	1

Table S2. Serine protease (SP) genes and representative transcripts expressed in the habu venom glands.

Gene	Name	Gene Model ID	Transcripts (Representative Site)	No. of Transcript Valiants in Venom Gland
svSP01	TLf1/flavoxobin	habu1_s7597_g18190a	pb016481_c628_f19p22_1897 pb016481_c809_f3p8_1564 pb016481_c386030_f8p29_3626 pb016481_c410809_f2p9_4011 pb016481_c402832_f1p16_3015 pb016481_ c581593_f1p4_3745 pb016481_ c768983_f1p7_7228	7
svSP02	TLf2		pb016481_ c578021_f1p16_2130 pb016481_ c240059_f1p15_2688 pb016481_ c53649_f1p1_1022 pb016481_ c1002973_f1p2_969 pb016481_ c79772_f1p2_185 pb016481_ c735101_f3p4_5942	6
svSP03	TLf3	habu1_s7597_g18190b	pb016481_ c254265_f2p2_2454 pb016480_ c578021_f1p16_2130 pb016481_c663_f11p12_1578 pb016481_ c746_f5p6_1124 pb016481_ c552114_f3p6_3482 pb016480_ c424167_f1p1_3699	6

svSP04	habu1_s6789_g17480	pb016480_ c54903_f1p3_2873 pb016481_ c389086_f1p3_2991 pb016481_c222250_f5p18_2824 pb016481_ c401044_f1p7_2131 pb016481_ c249172_f1p8_3106 pb016481_ c185672_f1p0_2196 pb016481_c575210_f1p3_3845 pb016480_c167245_f1p2_3915	8
svSP05	habu1_s6789_g17481a	pb016480_c5437_f4p4_1540 pb016480_c639_f14p4_1553 pb016481_c237569_f1p19_1959 pb016481_c720_f4p14_1676 pb016481_c79772_f1p2_1851 pb016481_c422554_f7p13_3481	6
svSP06	habu1_s4106_g13431a	pb016481_c782298_f2p3_6239 pb016481_c222442_f2p0_2603 pb016481_c3562_f3p0_1298 pb016481_c734984_f12p12_6638 pb016480_c230851_f5p19_8340 pb016481_c579606_f1p14_5087 pb016480_c124259_f1p4_2664	7
svSP07	habu1_s4106_g13431b	pb016481_c395211_f1p12_2348 pb016481_c10307_f1p3_1733 pb016480_c51061_f1p2_1886 pb016481_c9384_f1p12_1226 pb016481_c241758_f2p27_2330 pb016481_c244787_f3p7_23	9

			21<br>pb016481_c423385_f1p9_4252<br>pb016481_c576381_f1p1_4316<br>pb016481_c575991_f1p3_5233	
svSP08		habu1_s6789_g17481b	pb016481_c1327_f9p6_1578<br>pb016481_c572213_f1p3_1114<br>pb016481_c89829_f1p1_1252	3
svSP09		habu1_s7597_g18190c	pb016481_c243922_f1p5_2691<br>pb016481_c223329_f2p3_1902<br>pb016481_c1003251_f1p1_815	3
svSP10		habu1_s4106_g13432	pb016481_c944_f4p2_1299<br>pb016481_c735101_f3p4_5942<br>pb016481_c750442_f1p4_6280	3
svSP11	Jerdonobin-II like, GPV-PA	habu1_s22023_g22469	pb016481_c240059_f1p15_2688<br>pb016480_c344_f6p31_1937<br>pb016481_c552365_f2p5_3547	3
